# Seroprevalence and risk factors of small ruminant brucellosis in Jabodetabek, Indonesia

**DOI:** 10.14202/vetworld.2025.888-895

**Published:** 2025-04-19

**Authors:** Eny Martindah, Susan Maphilindawati Noor, Sutiastuti Wahyuwardani, Wasito Wasito, Dyah Ayu Hewajuli, Riyandini Putri, Sri Suryatmiati Prihandani, Andriani Andriani, Sumirah Sumirah, Andi Mulyadi, Naila Arsy Kun Azizah

**Affiliations:** 1 Research Centre for Veterinary Science, The National Research and Innovation Agency, Cibinong Science Centre, Jl. Raya Jakarta-Bogor KM. 46, Bogor, 16911, Indonesia; 2 Department of Remote Sensing, Faculty of Geography, Universitas Gadjah Mada, Bulaksumur, Yogyakarta, 55281, Indonesia

**Keywords:** brucellosis, goats, Indonesia, risk factors, seroprevalence, sheep, zoonotic diseases

## Abstract

**Background and Aim:**

Brucellosis, caused by *Brucella* spp., is a zoonotic disease of major public health and economic significance. In Indonesia, surveillance efforts have predominantly focused on bovine brucellosis, leaving limited data on small ruminants despite their critical role as disease reservoirs. This study aims to estimate the seroprevalence of brucellosis in goats and sheep and to identify potential risk factors associated with its transmission in the Jabodetabek region, Indonesia.

**Materials and Methods:**

A cross-sectional study was conducted from May 2023 to November 2023, involving 18 herds of goats and sheep across Jakarta, Bogor, Depok, Tangerang, and Bekasi. A total of 665 blood samples (355 from goats and 310 from sheep) and 112 milk samples were collected. The samples were analyzed using the Rose Bengal Test, complement fixation test, and enzyme-linked immunosorbent assay. Descriptive statistics were used to estimate seroprevalence, and a Chi-square test was employed to evaluate risk factors. Odds ratios (ORs) with 95% confidence intervals (CIs) were calculated to assess associations between risk factors and seropositivity.

**Results:**

The herd-level seroprevalence was 66.67% (12/18), with a mean within-herd seroprevalence of 10.39% (95% CI: 7.21–13.57). The animal-level seroprevalence was 6.17% (41/665), with the highest rates observed in Bogor City (11.89%), followed by Bekasi (8.91%), East Jakarta (8.00%), and Tangerang (4.58%). Depok City had no positive cases in serum tests, though two cases were detected through milk ELISA. Mixed-species farms exhibited a significantly higher risk of infection than single-species farms (OR: 0.30, 95% CI: 0.14–0.66, p < 0.05). No significant difference was observed between goats and sheep (p > 0.05), nor between males and females (p = 0.84).

**Conclusion:**

This study highlights a high seroprevalence of brucellosis in small ruminants within Jabodetabek, with mixed-species farming identified as a major risk factor. The findings underscore the need for enhanced surveillance, control measures, and public health interventions to mitigate disease transmission.

## INTRODUCTION

Brucellosis is a bacterial zoonotic disease that significantly affects human health and livestock productivity, particularly in low- and middle-income countries [1]. The World Health Organization (WHO) has classified brucellosis as a neglected zoonotic disease, underscoring the urgent need for improved awareness, surveillance, and control measures [2]. The causative agent, *Brucella* spp., is a Gram-negative, non-spore-forming, facultative intracellular coccobacillus responsible for reproductive disorders in livestock and severe systemic illness in humans [3]. In Indonesia, *Brucella abortus* and *Brucella suis* are frequently reported in livestock, contributing to economic losses and public health risks [4, 5]. Among small ruminants, particularly sheep and goats, brucellosis results in reproductive failure, including abortion, stillbirth, and infertility, leading to substantial productivity losses [6]. In addition, *Brucella melitensis* – the most pathogenic species for humans – is primarily transmitted through direct contact with infected animals or consumption of contaminated animal products, making goats and sheep key reservoirs [2, 7].

Brucellosis in humans manifests as undulant fever, excessive sweating, fatigue, arthralgia, weight loss, and hepatosplenomegaly, which can result in persistent flu-like symptoms and chronic complications [8]. A retrospective study in Beijing from 2010 to 2021 reported that patients in the acute phase of brucellosis were significantly more likely to present with splenomegaly, whereas arthralgia was more prevalent in chronic cases [9]. The disease is endemic in many regions and is often spatially associated with small ruminants, which act as primary reservoirs [10].

In Indonesia, brucellosis has a significant economic impact, with estimated annual losses of Indonesian Rupiah (IDR) 3.6 trillion, accounting for approximately 1.8% of the total value of livestock assets [11]. These losses arise from direct factors such as reduced reproductive performance, decreased milk production, and lower meat yields, as well as indirect consequences, including veterinary costs and trade restrictions [12]. As a zoonotic disease, brucellosis also leads to decreased productivity among farmers and agricultural workers due to illness. Recognizing its economic and public health significance, the Indonesian government has designated brucellosis as a strategic livestock disease requiring control under the Decree of the Minister of Agriculture No. 4026/Kpts/OT.140/2013.

Despite its economic and zoonotic importance, research on small ruminant brucellosis in Indonesia remains limited. Most epidemiological studies and surveillance efforts have predominantly focused on *B. abortus* in cattle, with small ruminants receiving minimal attention. Consequently, there is a critical gap in data regarding the seroprevalence, distribution, and risk factors of brucellosis in goats and sheep, particularly in high-density regions such as Jabodetabek. The lack of comprehensive epidemiological studies hinders the development of targeted interventions, limiting effective disease control strategies. Addressing this knowledge gap is essential for mitigating the impact of brucellosis on both animal health and public health.

This study aims to estimate the seroprevalence of brucellosis in small ruminants (goats and sheep) in the Jabodetabek region and to investigate potential risk factors contributing to its transmission. The findings will provide a foundational understanding necessary for designing targeted surveillance, control measures, and policy recommendations to reduce the burden of brucellosis in Indonesia.

## MATERIALS AND METHODS

### Ethical approval

This study underwent a thorough ethical review and received approval from the Research Ethics Committee under Experimental Animal Research, the National Research and Innovation Agency, Indonesia. Ethical clearance was granted under Certificate No. 065/KE.02/SK/04/2023 on April 11, 2023.

### Study period and location

A cross-sectional study was conducted from May 2023 to November 2023 in the Jabodetabek region, an acronym representing Jakarta, Bogor, Depok, Tangerang, and Bekasi. This area encompasses Jakarta’s special capital regions as well as parts of West Java and Banten provinces. Study sites were selected based on the high density of goats and sheep, the presence of both commercial and small-scale farms, and the willingness of farmers to participate.

### Sample collection

The number of samples collected was proportionate to the goat and sheep population density in each location. A simple random sampling method was employed to select animals, ensuring a representative sample size. Key risk factors – including species, age, sex, farm type, and geographical location – were documented during data collection for subsequent analysis.

This study considered the seroprevalence of brucellosis in small ruminants from a previous study by Martindah *et al*. [13] of 21.30% with a 95% confidence level and 5% desired precision. Based on these assumptions, the number of goats and sheep to be included in the study was determined using the following formula: n = Z² × P_exp_ (1−P_exp_)/d², where n is the required sample size, d is absolute precision/the margin of error (0.05), Z = 1.96 is the multiplier from the normal distribution for a 95% confidence interval (CI), P_exp_ is the expected seroprevalence from previous study, and P_exp_ is the probability of not having the disease [14]. The calculation suggested that at least n samples = 304 blood samples from small ruminants should be collected. In total, 665 blood samples (355 goats and 310 sheep) were collected from 18 farms for this study, along with 112 milk samples from goats. We collected goat milk samples to address a critical public health issue because goat milk is commonly sold, and many communities in Indonesia still consume raw goat milk due to perceived health benefits and nutritional value.

### Laboratory analysis

Blood samples were taken 5 mL aseptically from the jugular vein for serum collection. Sera were collected immediately in Eppendorf tubes and stored at 4°C before testing. All sera were tested with the Rose Bengal Test (RBT) (ID.vet, France), complement fixation test (CFT) (ID.vet), and indirect enzyme-linked immunosorbent assay (i-ELISA). ELISA (ID.vet). The RBT, CFT, and i-ELISA results were interpreted in parallel, where an individual or flock was considered seropositive if either test yielded a positive result. Conversely, a result was considered negative if all tests were negative. A herd with at least one animal that was seropositive for RBT or CFT was considered a positive flock. In addition, antibodies in milk samples were detected using i-ELISA.

### Statistical analysis

The data pertaining to individual animals were tabulated into a Microsoft Excel spreadsheet 2021 (Microsoft, Washington, USA). We determined animal seroprevalence using the following formula [14]:


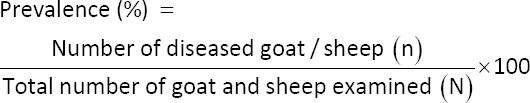


Herd-level seroprevalence was calculated for descriptive purposes using two approaches [14]:

(1) The simple mean of individual herd seroprevalence, calculated as the average seroprevalence across herds, treating each herd equally regardless of sample size:


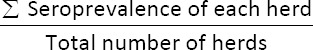


The proposed method provides an unweighted estimate of the average seroprevalence among herds.

(2) Population-weighted seroprevalence, which accounts for differences in herd sample sizes:


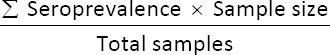


This method ensures that larger herds contribute proportionally to the overall seroprevalence estimate.

The 95% CIs for both estimates were calculated using standard error calculations.

Risk factor analysis, however, was conducted at the individual animal level, where associations between seroprevalence and potential risk factors – including location, farm type, species, age, and sex – were assessed using the Chi-square (χ^2^) test [15]. The strength of the associations between risk factors and seroprevalence was calculated using odds ratios (OR) with a 95% CI, and the statistical significance level was set at p < 0.05. The association between variables was considered significant when p < 0.05.

## RESULTS

### Herd-level seroprevalence

The study involved 18 farms consisting of 11 (61.11%) goat herds, 3 (16.67%) sheep herds, and 4 (22.23%) mixed herds (goat and sheep). A total of 665 blood samples, comprising 355 (53.38%) goat and 310 (46.62%) sheep blood samples, were collected, along with 112 milk samples from goats. The herd-level seroprevalence for small ruminant farms was 66.67% (12 out of 18; 95% CI: 43.75–83.72), as estimated through RBT and CFT testing. The seroprevalence of brucellosis among the herds varied widely, ranging from 0% to 33.33%. The mean herd-level seroprevalence, calculated as a simple average of individual herd seroprevalences, was 10.39% (95% CI, 7.21–13.57). However, since this method gives equal weight to all herds regardless of sample size, the population-weighted seroprevalence was 7.52% (95% CI, 5.51–9.52). This highlights differences in disease exposure between herds, with some having no positive cases while others showing high infection rates (Table 1).

**Table 1:** Herd-level seroprevalence of brucellosis among small ruminant herds (goats and sheep) in Jabodetabek, Indonesia.

Location	Herd number	Species within the herd	Seroprevalence (%) (numbers seropositive/total sample)
East Jakarta	1	Goat	11.43 (4/35)
East Jakarta	2	Goat	11.76 (2/17)
East Jakarta	3	Goat	6.25 (1/16)
East Jakarta	4	Goat	0 (0/7)
East Jakarta	5	Goat	0 (0/4)
East Jakarta	6	Goat	4.76 (1/21)
Bogor City	7	Mixed (goat and sheep)	14.71 (10/68)
Bogor City	8	Sheep	19.35 (6/31)
Bogor City	9	Goat	25 (1/4)
Bogor City	10	Goat	0 (0/19)
Bogor City	11	Goat	0 (0/21)
Depok City	12	Goat	0 (0/78)
Depok City	13	Sheep	0 (0/90)
Tangerang City	14	Sheep	3.61 (3/83)
Tangerang City	15	Mixed (goat and sheep)	5.71 (4/70)
Bekasi Regency	16	Goat	33.33 (1/30)
Bekasi Regency	17	Mixed (goat and sheep)	5.00 (1/20)
Bekasi Regency	18	Mixed (goat and sheep)	13.73 (7/51)
Total			0–33.33% (41/665)
Simple mean (herd-level)			10.39%; (95% CI, 7.21–13.57)
Weighted mean (population-level)			7.52% (95% CI, 5.51–9.52)

### Animal-level seroprevalence and risk factors

**Figure 1:**
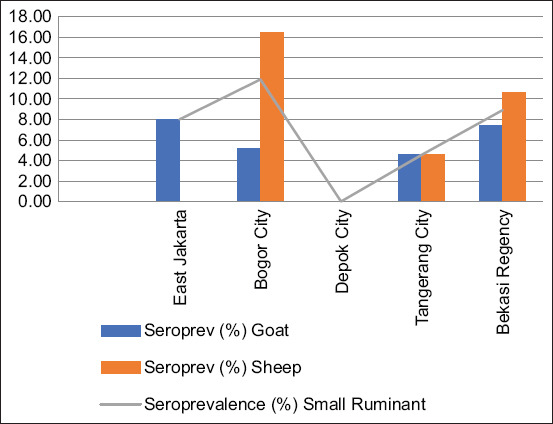
Seroprevalence of brucellosis reactor (+) in goats and sheep in Jabodetabek, Indonesia.

Table 2 shows 355 goat sera samples tested. Of these, 18 were seropositive for *Brucella* spp., representing a seroprevalence rate of 5.07% (95% CI: 3.23–7.87). Among the 310 sheep sera samples tested, 23 were seropositive, with a seroprevalence rate of 7.42% (95% CI: 4.99–10.89). There was no significant difference in seroprevalence between goats and sheep in the Jabodetabek area (χ^2^ = 0.18, p > 0.05). Sera samples were also tested using ELISA, and those not showing seropositivity were deemed negative for *Brucella* spp. The overall seroprevalence of brucellosis in small ruminants in the Jabodetabek area was 6.17% (95% CI: 4.58–8.26). The highest seroprevalence was observed in Bogor City (11.89%), followed by Bekasi Regency (8.91%), East Jakarta (8%), and Tangerang City (4.58%). Sera samples from Depok City did not detect positive brucellosis with RBT and CFT tests. Figure 1 shows the seroprevalence brucellosis in goats and sheep in Jabodetabek. Of the 112 milk goat samples tested with i-ELISA, 4/112 (3.57%) were positive for brucellosis, including two samples from Depok City in which serum tests were negative (Table 3).

**Table 2:** Seroprevalence of brucellosis in goats and sheep in Jabodetabek, Indonesia.

Location	Goats		Sheep		Overall seroprevalence (%); (95% CI)
	
	Seroprevalence (%)	95% CI	Seroprevalence (%)	95% CI	
East Jakarta	8/100 (8.00)	4.11-15.00	NA	NA	8/100 (8.00); (4.11–15.00)
Bogor City	3/58 (5.17)	1.77-14.14	14/85 (16.47)	10.07-25.77	17/143 (11.89); (7.56–18.21)
Depok City	0/78 (0.00)	0	0/90 (0.00)	0	0/168 (0.00); (0)
Tangerang City	3/65 (4.62)	1.58-12.71	4/88 (4.55)	1.78-11.11	7/153 (4.58); (2.23–9.14)
Bekasi Regency	4/54 (7.41)	2.92-17.55	5/47 (10.64)	4.63-22.59	9/101 (8.91) (4.76–16.67)
Total	18/355 (5.07)	3.23-7.87	23/310 (7.42)	4.99-10.89	41/655 (6.17); (4.58–8.26)

CI=Confidence interval, NA = not available (no sheep blood samples were obtained from East Jakarta)

**Table 3:** Result of milk samples tested for brucellosis.

No.	Location	Number of goat milk tested (n)	ELISA test positive (%)
1.	Bogor	24	0
2.	Jakarta	18	1/18 (5.56)
3.	Bekasi	16	1/16 (6.25)
4.	Tangerang	NA*	NA*
5.	Depok	54	2/54 (3.70)
Total		112	4/112 (3.57)

* NA=No milk samples were obtained from Tangerang. ELISA=Enzyme-linked immunosorbent assay

The results in Table 4 show that the odds of brucellosis seroprevalence were slightly lower in sheep farms than in mixed farms (OR < 1). Regarding individual risk factors associated with brucellosis in small ruminants, notable differences were observed in mixed farms, which have a significantly higher risk of brucellosis than goat and sheep farms (OR= 0.30, 0.14–0.66); p < 0.05. In contrast, no significant differences were observed between goat and mixed farms (OR: 0.80, 95% CI: 0.31–2.06, p = 0.81), indicating similar exposure risks between these farm types. Regarding age, older animals (≥2 years) had a higher seroprevalence than younger animals (<2 years); however, the difference was not statistically significant (OR: 0.60, 95% CI: 0.31–1.16, p = 0.15). Sex was not a significant risk factor, with no difference in brucellosis seroprevalence between male and female animals (OR: 0.93, 95% CI: 0.43–1.99, p = 0.84).

**Table 4:** Individual risk factors associated with brucellosis seroprevalence in small ruminant herds in Jabodetabek, Indonesia.

Risk factor	Category	Total (%)	Seroprevalence (%)	OR	95% CI	p-value
Farm type	Mixed farm	209 (31.43)	23/209 (11.00)	Reference	-	-
	Goat farm	252 (37.89)	9/252 (3.57)	0.80	0.31–2.06	0.81
	Sheep farm	204 (30.68)	9/204 (4.41)	0.30	0.14–0.66	0.003
Species	Goats	355 (53.38)	18/355 (5.07)	Reference	-	-
	Sheep	310 (46.62)	23/310 (7.42)	1.50	0.79–2.84	0.27
Age	<2 years	320 (48.12)	15/320 (4.69)	Reference	-	-
	>2 years	345 (51.88)	26/345 (7.54)	0.60	0.31–1.16	0.15
Sex	Female	527 (79.25)	32/527 (6.07)	Reference	-	-
	Male	138 (20.75)	9/138 (6.52)	0.93	0.43–1.99	0.84

Mixed farms refer to farms raising both goats and sheep together. Goats, animals aged <2 years, and females were used as reference groups for species, age, and sex comparisons. OR=Odds ratio, CI=Confidence interval

### Spatial distribution of seroprevalence brucellosis in small ruminants of Jabodetabek, Indonesia

**Figure 2:**
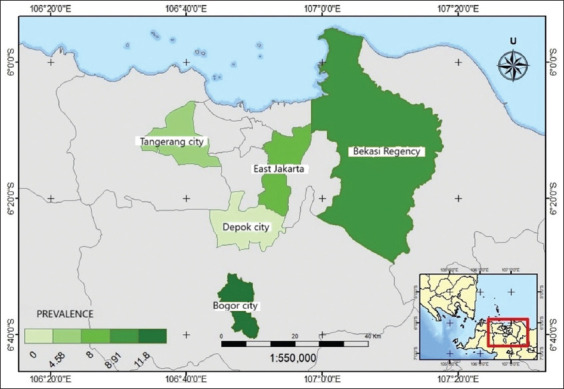
The spatial distribution of brucellosis seropositive small ruminants in Jabodetabek. Spatial data were obtained from the official Geospatial Information Agency of Indonesia. Website (Regional Boundary Data), and processed using ArcMap version 10.8. (https://tanahair.indonesia.go.id/).

Figure 2 illustrates the seroprevalence of brucellosis in small ruminants, showing varying seroprevalence rates across the study regions. This highlights the spatial diversity of the distribution of brucellosis in small ruminants.

## DISCUSSION

Brucellosis is a zoonosis that causes abortion in naturally infected small ruminants and is an important public health problem in many countries. This study provides valuable insights into the seroprevalence and risk factors of brucellosis in small ruminants in the Jabodetabek area of Indonesia. This work appears to be the first extensive study of its kind on the seroprevalence of small ruminant brucellosis in the study area in terms of sample size and coverage area. The overall seroprevalence of 6.17% (95% CI: 4.58–8.26) was recorded at the individual animal level, indicating a significant presence of brucellosis in the region, with variations across different locations. This seroprevalence is comparable to the global small ruminant brucellosis seroprevalence of 6.2% reported by Li *et al*. [16], with Africa reporting the highest at 8.5%, Gompo *et al*. [17] reported 15% and 1.1% seroprevalence in sheep and goats, respectively, in Nepal, and 5.21% in Ethiopia reported by Debano [18], suggesting that the Jabodetabek area faces similar challenges to other regions.

The herd level was examined to highlight the potential impact of *Brucellosis* spp. on goats and sheep, especially the economic consequences for smallholder farmers. The overall seroprevalence of brucellosis was estimated using two approaches [14]: Simple mean of individual herd seroprevalence and population-weighted seroprevalence. The simple mean treats each herd equally, providing insight into herd-level variability, whereas the weighted approach accounts for herd size, offering a more representative estimate at the population level. The difference between these estimates (10.39% vs. 7.52%) suggests that smaller herds may have had higher seroprevalence rates, which could indicate different risk factors across herd sizes. Reporting both values allows for a more comprehensive understanding of disease distribution and allows for comparisons with studies using similar methodologies.

The herd-level seroprevalence of 66.67% (95% CI: 43.75–83.72) in 12 out of 18 herds is particularly concerning, suggesting that brucellosis is widespread among the small ruminant population in the study area. This finding is consistent with prior studies. For example, research conducted in Qatar reported a flock-level seroprevalence ranging from 19.3% to 30.5% [19], in Ethiopia 39.74% [20], in Iran 11% seroprevalence among herds [21], and in Nepal, 31.6% was reported from sheep farm seropositive to brucellosis [17]. However, Ntirandekura *et al*. [22] reported that the herd level of seroprevalence brucellosis in small ruminants in Tanzania was 6.9% lower. This herd-level seroprevalence highlights the global nature of this issue in livestock management, highlighting the necessity for targeted interventions at the farm level to control the transmission of brucellosis.

The present study also revealed that the odds of brucellosis seroprevalence in goats were slightly lower than in sheep flocks (OR < 1); however, the result is not statistically significant (p > 0.05). This indicates that there is no strong evidence that goat farms have brucellosis rates that differ from those of sheep farms. Interestingly, although there was no statistically significant difference in seroprevalence between goat farms (3.57%) and sheep farms (4.41%), mixed farms showed a significantly higher risk of brucellosis than single-species farms. This finding aligns with a study by Diaz Aparicio [23], who suggested that interspecies transmission can occur, potentially explaining the higher risk in mixed farms. Keeping multiple species together may increase the risk of brucellosis transmission, possibly due to increased opportunities for cross-species infection or differences in management practices on mixed farms. A study in Thailand [24] suggested that larger flocks (including mixed farms) were linked with a higher incidence of brucellosis, indicating that mixed farming practices may contribute to the transmission of the disease. The variability in brucellosis seroprevalence across regions necessitates a control strategy based on local epidemiological data.

According to Saeed *et al*. [25], older small ruminants are more likely to test positive for *Brucella* infections than younger ones, suggesting that age is a critical risk factor for brucellosis. In the present study, although older animals (≥2 years) had higher seroprevalence than younger animals (<2 years), the difference was not statistically significant (OR: 0.60, 95% CI: 0.31–1.16, p = 0.15). Our study found no statistically significant association between age and brucellosis seroprevalence, which contradicts previous research. For instance, a study in an agribusiness farm in a semi-urban area of Bogor found that brucellosis reactor positivity was significantly higher in older animals (over 2 years) than in younger animals (0–2 years) [13]. Similarly, Dosa *et al*. [26] observed a higher seroprevalence in older animals (OR = 0.05, 95% CI = 0.03–0.07) than in young animals. The presence of older sheep, especially those older than 1.5 years, increases the risk of infection due to the potential for cumulative exposure to the pathogen [20]. In addition, Promsatit *et al*. [24] repoted a study in Thailand that animals aged 12-24 months had the highest apparent seroprevalence (43.90%). A study in India by Behera *et al*. [27] also reported that increasing age is a major risk factor for brucellosis. This discrepancy may be due to local factors, such as management practices, biosecurity measures, or the specific strains of *Brucella* present in the region.

The absence of sex-based differences in seroprevalence in the present study (OR: 0.93, 95% CI: 0.43–1.99, p = 0.84) suggests that male and female animals in our study population are equally susceptible to infection. This finding aligns with previous research by Behera *et al*. [27] and indicates that sex was not a significant risk factor for brucellosis seropositivity in this study. However, some studies have reported a higher seroprevalence in females, due to reproductive factors [7]. Although brucellosis in females can lead to stillbirths, infertility, abortion, and reduced product quality, all of which can significantly cause economic losses [28], our data did not support a sex-based difference in infection risk.

The spatial distribution of brucellosis seroprevalence, with the highest rates in Bogor City (11.89%) and the lowest in Depok City (0%), highlights the importance of considering geographic factors in disease control strategies. These variations may be influenced by factors such as animal movement patterns, farm management practices, and environmental conditions that differ across locations. Similar spatial variations have been observed in other studies, such as the regional differences noted by Li *et al*. [16] in their global analysis of sheep brucellosis. The spatial distribution map developed in this study provides a valuable tool for guiding future disease control efforts, similar to approaches used in other regions [22]. This approach can help prioritize interventions in high-prevalence areas and inform the development of region-specific control strategies, considering local epidemiological patterns and risk factors.

The detection of brucellosis in milk samples, including samples from Depok City, where serum tests were negative, emphasizes the importance of using multiple diagnostic methods for comprehensive surveillance. The study ensures robust detection of brucellosis seroprevalence, including specific insights from milk samples, contributing to additional positive cases that other tests missed. This finding also raises concerns about the potential for zoonotic transmission through contaminated milk products, a risk highlighted by the WHO [2] in its classification of brucellosis as a neglected zoonotic disease.

The economic impact of brucellosis, estimated at IDR 3.6 trillion in Indonesia [11], underscores the urgent need for effective control measures. This significant burden of economic and public health risks calls for a One Health approach to brucellosis control that integrates animal health, public health, and environmental considerations [6]. The findings highlight the importance of implementing comprehensive surveillance programs integrated with multiple diagnostic methods, including serological tests and milk sampling. Such programs would provide a more accurate picture of the disease’s prevalence and distribution, thereby addressing the potential underestimation of brucellosis [3].

## CONCLUSION

This study provides the first extensive investigation into the seroprevalence and risk factors of small ruminant brucellosis in the Jabodetabek region of Indonesia, addressing a significant knowledge gap in the epidemiology of the disease in non-bovine livestock. The findings highlight a high herd-level seroprevalence (66.67%) and an animal-level seroprevalence of 6.17%, with geographical variations indicating Bogor City as the highest-risk area. The study further identifies mixed-species farming as a critical risk factor, demonstrating significantly higher odds of brucellosis transmission compared to single-species farms. While no significant differences were observed between goats and sheep in terms of seroprevalence, spatial analysis indicates the potential for localized disease hotspots.

This study is novel in its focus on small ruminants in an urban-agricultural interface, a setting often overlooked in brucellosis research. By utilizing a combination of serological tests (RBT, CFT, and ELISA), the study ensures high diagnostic reliability. In addition, the use of spatial analysis techniques provides a valuable epidemiological perspective, allowing for targeted intervention planning. The multivariable logistic regression model strengthens the study’s findings by controlling for confounding factors, thereby offering a more precise assessment of disease risk factors.

Despite its strengths, the study has certain limitations. The cross-sectional design captures seroprevalence at a single time point, limiting the ability to assess temporal trends or causal relationships. While serological tests provide reliable detection of exposure, they do not distinguish between active and past infections. Potential underreporting may have occurred due to variability in herd management practices and willingness of farmers to participate. In addition, molecular characterization of *Brucella* species was not conducted, which could have provided insights into strain diversity and transmission dynamics.

Future research should focus on longitudinal studies to assess disease progression and transmission dynamics over time. Molecular and genomic studies on *Brucella* species circulating in small ruminants will be crucial for understanding strain variation and zoonotic potential. In addition, socioeconomic assessments should be integrated to evaluate the economic impact of brucellosis on smallholder farmers and the cost-effectiveness of potential control strategies, such as vaccination and biosecurity measures. A One Health approach, integrating veterinary, public health, and environmental surveillance, is recommended to mitigate the burden of brucellosis in Indonesia. This study provides a strong foundation for targeted disease control strategies and reinforces the necessity of enhanced surveillance, farmer education, and policy interventions to reduce the public health and economic impact of brucellosis in small ruminants.

## AUTHORS’ CONTRIBUTIONS

EM and SMN: Conceived, designed, and coordinated the study. SMN, SW, EM, RP, WW, DAH, SSP, AA, SS, and AM: Supervised the field sampling, data collection, and laboratory work. EM, SMN, RP, SW, SSP, AA, and NAKA: Data entry, data analysis and interpretation, and drafted the manuscript. EM, SMN, RP, SW, WW, and DAH: Reviewed and edited the manuscript. All authors have read and approved the final version of the manuscript.
